# Furanodiene Induces Endoplasmic Reticulum Stress and Presents Antiproliferative Activities in Lung Cancer Cells

**DOI:** 10.1155/2012/426521

**Published:** 2012-08-08

**Authors:** Wen-Shan Xu, Yuan-Ye Dang, Jia-Jie Guo, Guo-Sheng Wu, Jin-Jian Lu, Xiu-Ping Chen, Yi-Tao Wang

**Affiliations:** State Key Laboratory of Quality Research in Chinese Medicine and Institute of Chinese Medical Sciences, University of Macau, Avenue Padre Toma's Pereira S.J., Taipa, Macau 999078, China

## Abstract

Furanodiene (FUR) is a natural terpenoid isolated from *Curcumae Rhizoma*, a well-known Chinese medicinal herb that presents antiproliferation activities in several cancer cell lines. In this study, we demonstrated that FUR concentration dependently inhibits the cell proliferation of A549, NIH-H1299, and 95-D lung cancer cells. **β**-elemene, another terpenoid isolated from *Curcumae Rhizoma*, exhibited weaker antiproliferative effects in A549 and NIH-H1299 cells and activities similar to FUR in 95-D cells. FUR significantly inhibited colony formation in A549 and 95-D cells and upregulated both the mRNA and protein expression levels of binding immunoglobulin protein (BIP) and C/EBP homologous protein (CHOP), indicating that endoplasmic reticulum (ER) stress is induced. FUR treatment led to the accumulation of CHOP in the nucleus, which further confirms induction of ER stress. Furthermore, combined treatment of FUR with paclitaxel showed significant synergetic activities in NIH-H1299 and 95-D cells, suggesting its potential roles in combination therapy. These findings provide a basis for the further study of the anticancer effects *in vivo* and the internal mechanisms of FUR.

## 1. Introduction

**Figure 1:**
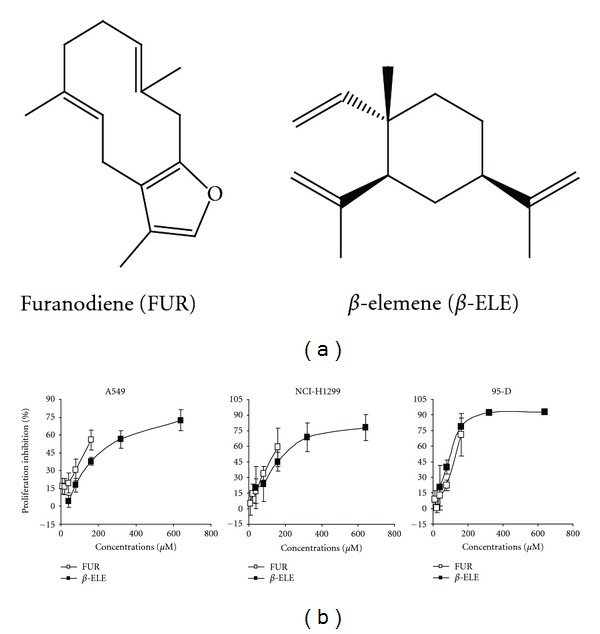
Comparison of FUR and *β*-ELE on lung cancer cell proliferation. (a) Chemical structure of FUR and *β*-ELE, (b) cells were treated with FUR or *β*-ELE for 48 h, after which cell proliferative inhibition was tested by MTT assay.

Lung cancer is a leading type of cancer worldwide and no effective chemotherapy agents for it have yet been developed [1]. Determination of anticancer compounds from traditional herbs may offer new options for lung cancer treatment [2–6]. *Curcumae Rhizoma *(or Ezhu in Chinese), a commonly used traditional Chinese medicinal herb, has displayed wide and diverse medicinal value for almost one thousand years, including removal of blood stasis and pain alleviation. The herb has been widely prescribed to treat cardiovascular diseases and cancer in Chinese clinical practice [7]. Besides, this herb is also included in some of the traditional Chinese medicine compound recipe, such as compound recipe Ezhu, for cancer treatment. Furanodiene (FUR, Figure 1(a)), a sesquiterpene isolated from *Curcumae Rhizoma*, inhibits the growth of several human cancer cell lines, including leukemia HL-60 cells, cervical cancer HeLa cells, prostatic cancer PC3 cells, larynx carcinoma Hep-2 cells, gastric carcinoma SGC-7901 cells, and fibrosarcoma HT-1080 cells and also inhibits the growth of uterine cervical and sarcoma 180 tumors in mice [8, 9]. However, no reports have yet been found on its anti-cancer effects in lung cancer cells and limited studies on its anti-cancer mechanisms are available. The current consensus is that FUR activates p38 mitogen-activated protein kinase and inactivates extracellular signal-regulated kinase signaling, inducing cell cycle arrest and caspase-dependent apoptosis, and so on [9, 10]. 

Elemene is a mixture isolated from *Curcumae Rhizoma* and is mainly composed of *β*-elemene (*β*-ELE, Figure 1(a)) [2]. Elemene exhibits various anti-cancer activities [2] and has already been approved as a new anti-cancer drug by the State Food and Drug Administration of China. However, *Curcumae Rhizoma *contains much less *β*-ELE than FUR [11]. Therefore, determination of whether or not FUR has anti-cancer activities similar to that of *β*-ELE could promote the use of the herb.

This study aims to (1) compare the anticancer effects of *β*-ELE and FUR in human lung cancer cells, (2) explore the possible anti-cancer mechanisms of FUR, and (3) detect the combined therapeutic effects of FUR with common chemotherapeutic agents. We found that FUR exhibits anti-cancer activities stronger than or similar to those of *β*-ELE in three tested lung cancer cell lines and that it retards colony formation and triggers endoplasmic reticulum stress (ER stress) in A549 and 95-D cells. The effects of the combined treatment of FUR and doxorubicin (DOX) or paclitaxel (TAX) were also investigated.

## 2. Materials and Methods

### 2.1. Materials

FUR was separated and purified from the essential oil of *Curcuma wenyujin* by silica-gel-column separation [12] and the purity of FUR (>96%) was tested by HPLC-DAD [11]. *β*-ELE was purchased from WAKO (Japan). The stock concentration of *β*-ELE and FUR was 320 mM and 160 mM, respectively. DOX and TAX were obtained from Sigma (St. Louis, MO, USA) and the stock concentration was 4 mM and 1 mM, respectively. 3-[4,5-Dimethyl-2-thiazolyl]-2,5-diphenyl tetrazolium bromide (MTT) was purchased from Amersham (Sweden).

### 2.2. Cell Culture

The human lung cancer cell lines A549 (ATCC, Rockville, MD, USA), NCI-H1299 (ATCC), and 95-D (The Cell Bank of Type Culture Collection of Chinese Academy of Sciences, Shanghai, China) were cultured in RPMI 1640 medium supplemented with 10% fetal bovine serum (GIBCO BRL, Carlsbad, CA, USA), 100 U/mL penicillin, and 100 mg/mL streptomycin and were grown in an incubator with 5% CO_2_ at 37°C.

### 2.3. MTT Assay

Exponentially growing A549, NCI-H1299, and 95-D cells were planted into 96-well plates, and then treated with serial concentrations of FUR or *β*-ELE for 48 h after adhesion. For the combinational treatment, FUR, DOX, and TAX were simultaneously added into the wells and treated for 48 h according to the combined treatment design of the experiment. Cell proliferation was determined by addition of 1mg/mL MTT-containing medium for 4 h, addition of 100 *μ*L DMSO to solubilize the formazan, and shaking for 10 min in the dark. The absorbance at 570 nm was recorded using a multilabel counter (Perkin Elmer, Singapore).

### 2.4. Observation of Morphological Changes

A549 and 95-D cells were seeded into 6-well plates and treated with the indicated concentration of FUR for 24 h. The cellular morphology was observed using an AxioCam HRC CCD camera (Carl Zeiss).

### 2.5. Colony Formation Assay

A549 and 95-D cells were treated with serial concentrations of FUR for 24 h (200 nM TAX was used as a positive control) and then suspended and reseeded into 6-well plates at a density of 200 cells per well (A549) or 1000 cells per well (95-D) after treatment. Cells were subsequently fixed using 4% paraformaldehyde and stained with crystal violet staining solution (Beyotime Institute of Biotechnology, China) after one-week incubation. Typical images were captured using an ordinary NIKON camera. 

### 2.6. Real-Time PCR

A549 and 95-D cells were treated with 60 *μ*M and 80 *μ*M FUR for 12 h and the total RNA was extracted using the RNeasy mini kit (Qiagen, 74106) according to the manufacturer's protocol. The primer sequences were as follows: 5′-TCCTATGTCGCCTTCACT-3′ (forward), 5′-ACAGACGGGTCATTCCAC-3′ (reverse) for binding immunoglobulin protein (BIP); 5CTGACCAGGGAAGTAGAGG-3′ (forward), 5′-TGCGTATGTGGGATTGAG-3′ (reverse) for C/EBP homologous protein (CHOP); 5′-CCATGGAGAAGGCTGGGG-3′ (forward); 5′-CAAATGTGTCATGGATGACC-3′ (reverse) for GAPDH. One milligram of total RNA was reverse transcribed using the high capacity cDNA reverse transcription kit with RNase inhibitor (Invitrogen, 4374966) and the cDNA template was amplified by PCR using the Fast SYBR Green Master Mix (Applied biosystems, 4385616). Thermal cycling was programmed according to the manufacturer's protocol. Gene expression was assessed by ΔΔCt method and mRNA levels of BIP and CHOP were normalized to those of GAPDH internal standard.

### 2.7. Western Blot Analysis

Cells were lysed in the lysis buffer, and the proteins of the lysates were quantified using a BCA protein assay kit (Pierce, Rockford, IL, USA). About 30 *μ*g of total proteins was subjected to SDS-PAGE, transferred onto nitrocellulose membranes, and then blocked with 5% nonfat milk in TBST (20 mM Tris, 500 mM NaCl, and 0.1% Tween-20) at room temperature for 2 h with rocking. The membranes were probed with specific primary antibodies against BIP and CHOP (Cell Signaling Technology, Beverly, MA, USA) overnight at 4°C. After washing with TBST three times for 15 min each, the membranes were incubated with horseradish-peroxidase-conjugated secondary antibodies (Santa Cruz Biotechnology Inc.) in TBST at room temperature for 1 h, and specific protein bands were visualized using an ECL advanced Western blot detection kit. Equal protein loading was verified through rehybridization of the membranes and reprobing with anti-*β*-actin antibody.

### 2.8. Immunocytochemical Labeling

A549 and 95-D cells were fixed with 4% formaldehyde in PBS at 37°C for 30 min, washed with PBS, and then permeabilized with 0.5% Triton X-100 in PBS for 20 min at room temperature after FUR treatment. Cells were washed in a blocking solution containing 5% BSA and 0.2% Triton X-100 and stored in the blocking solution at 4°C until labeling. Fixed cells were then incubated at 4°C with the specific antibody against CHOP in the blocking solution overnight. After three washes in the blocking solution, cells were incubated with goat anti-rabbit IgG in the blocking solution at 37°C for 1 h. After three washes, cells were incubated for 10 min at room temperature with Hoechst 33342. Cells were then mounted in a 90% glycerol-PBS mixture after three washes with PBS, after which they were visualized and photographed with an IX81 fluorescent microscope (Olympus, Japan).

### 2.9. Statistical Analysis

Combination index (CI) is well accepted for quantifying drug synergism based on the multiple-drug effect equation of Chou-Talalay [13, 14]. In the current study, CI values were tested for each concentration of FUR, DOX, or TAX, and the corresponding combination in cell proliferation assays using Calcusyn (Biosoft, Cambridge, United Kingdom). A CI lower than 0.9 indicates synergism, a CI of 0.9 to 1.10 indicates additive, and a CI higher than 1.10 indicates antagonism [15].

## 3. Results

### 3.1. Comparison of the Anticancer Effects of FUR and *β*-ELE on Lung Cancer Cells

*β*-ELE is an anti-cancer drug obtained from the same source as FUR [2, 11]; thus, the anti-proliferative activities of these two compounds in lung cancer cells were compared. Cells were treated with various concentrations of FUR or *β*-ELE for 48 h, and both compounds inhibited cell proliferation in a concentration-dependent manner, as detected by MTT assay (Figure 1(b)). *β*-ELE showed weaker anti-proliferative activities compared with FUR in A549 and NIH-H1299 lung cancer cells (Figure 1(b)) and MCF-7 breast cancer cells (data not shown) but exhibited effects similar to FUR in 95-D cells (Figure 1(b)). This result indicates that FUR has similar or even better anti-cancer potential compared with *β*-ELE.

### 3.2. FUR Inhibits Colony Formation in Lung Cancer Cells

**Figure 2:**
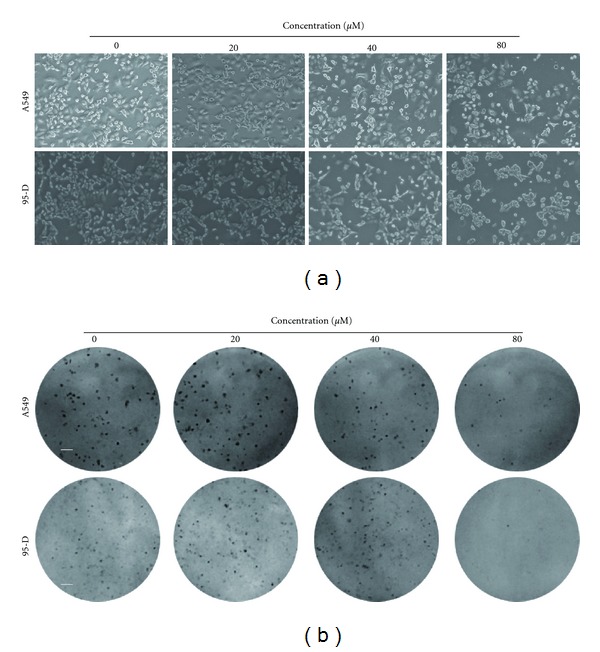
FUR inhibition on colony formation in lung cancer cells. (a) Cells were treated with various concentrations of FUR for 24 h, after which morphological changes were observed using an AxioCam HRC CCD phase contrast microscope. Bar: 20 *μ*m. (b) Cells were treated with various concentrations of FUR for 24 h. After treatment, cells were suspended and reseeded into 6-well plates at a density of 200 cells per well (A549) or 1000 cells per well (95-D) and then fixed and stained with 4% PFA and crystal violet after one week. The representative images of colony formation assay were obtained. Bar: 2 mm.

The colony formation potential of FUR on lung cancer A549 and 95-D cells after FUR treatment was determined. Cells were treated with serial concentrations of FUR for 24 h, and morphological changes after FUR treatment were observed using phase-contrast microscopy (Figure 2(a)). FUR decreased the percentage of adherent cells in a concentration-dependent manner in both cell lines. In addition, the colony formation assay was used to further investigate the anti-cancer potential of FUR. 200 nM TAX was used as a positive control. The number of colony foci decreased after treatment with 80 *μ*M FUR (Figure 2(b)) and TAX (data not shown) in both cell lines, consistent with the results of morphological changes (Figure 2(a)) and the MTT assay (Figure 1(b)).

### 3.3. Induction of ER Stress after FUR Treatment in Lung Cancer Cells

**Figure 3:**
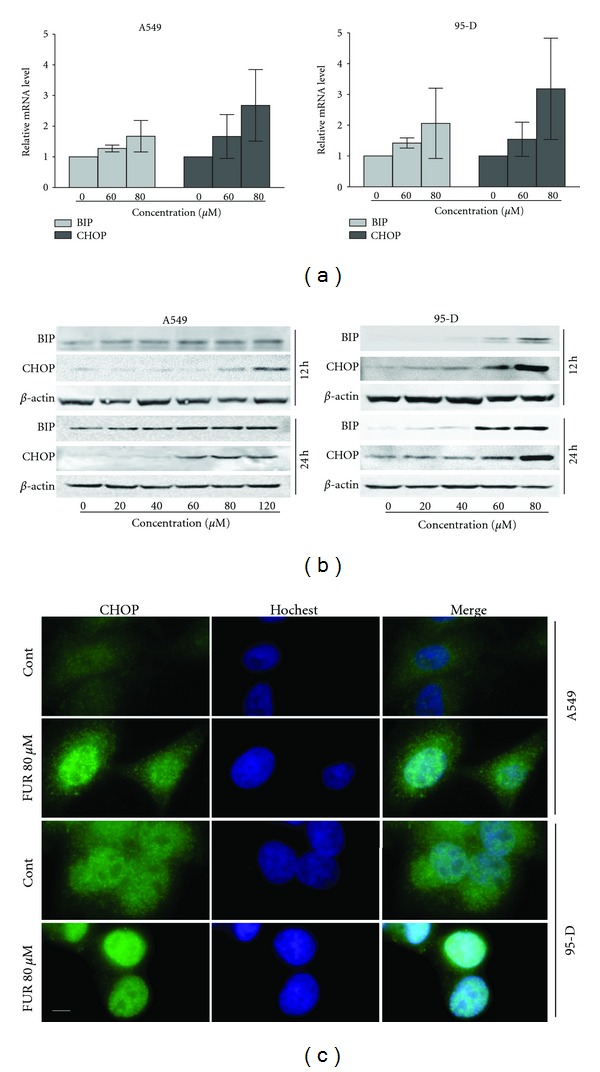
FUR induces ER stress in lung cancer cells. (a) Effect of FUR on the mRNA levels of BIP and CHOP in A549 and 95-D cells. Cells were treated with different concentrations of FUR for 12 h, and mRNA levels were determined by RT-PCR. (b) Effect of FUR on the protein levels of BIP and CHOP in A549 and 95-D cells. Cells were treated with different concentrations of FUR for 12 h or 24 h, and protein levels were determined by Western blot. (c) Immunocytochemical staining was conducted to detect the expression of CHOP in nuclei. Cells were treated with 80 *μ*M FUR for 12 h and the cells were fixed and stained with anti-CHOP antibody (green) and Hoechst 33342 (blue). Bar: 10 *μ*m.

The mechanisms by which FUR produces its anti-proliferative effects were also investigated in this study. Various compounds induce ER stress [16, 17], which can trigger a signal transduction network to induce cell proliferation inhibition. We therefore investigated whether or not FUR induces ER stress in lung cancer cells. Induction of BIP and CHOP in A549 and 95-D cells after 12 h of FUR treatment was first investigated because responses to ER stress are characterized in part by the increased transcription of BIP and induction of CHOP [18, 19].  Figure 3(a) shows that FUR induced an increase in BIP and CHOP at the mRNA level in both cell lines. Induction of BIP and CHOP at the protein level in A549 and 95-D cells after 12 h and 24 h of FUR treatment was also confirmed (Figure 3(b)). Furthermore, the numbers of CHOP foci also increased after FUR treatment for 12 h in both cell lines (Figure 3(c)) while CHOP foci were much more obvious in 95-D cells than those in A549 cells. In summary, FUR induces ER stress in A549 and 95-D lung cancer cells, which may contribute at least in part to its anti-cancer potential.

### 3.4. The Effects of FUR in Combination with DOX or TAX in Different Lung Cancer Cell Lines

**Figure 4:**
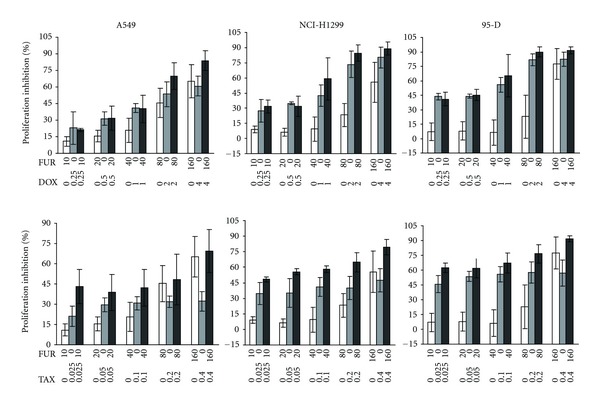
Combined cytotoxicity of FUR and DOX or FUR and TAX in three lung cancer cell lines. Cells in 96-well plates were treated with serial concentrations of FUR with or without the indicated DOX or TAX for 48 h. Cell proliferative inhibition was tested using MTT assay.

The anti-proliferative effects of FUR were further determined through the combined treatment of FUR and DOX or FUR and TAX on A549, NCI-H1299, and 95-D human lung cancer cell lines. Cells in 96-well plates were treated with serial concentrations of FUR with or without the indicated DOX or TAX for 48 h. The cell proliferative inhibition bars were shown in Figure 4. FUR, DOX, and TAX displayed cytotoxicity in a concentration-dependent manner against all three cancer cell lines. Much stronger anti-proliferation abilities were achieved after combinational treatment (except at low concentrations of DOX and FUR) compared with treatment with FUR, DOX or TAX alone. CI values were calculated using Calcusyn at fixed ratio concentrations of FUR and DOX or FUR and TAX.  Table 1 showed that FUR plus TAX presents obvious synergistic anti-cancer effects in NIH-H1299 and 95-D cells while this effect was weaker in A549 cells. FUR plus DOX also showed synergistic anti-cancer effects at high concentrations, whereas antagonism was observed at lower doses in all tested three cell lines (Table 1).

## 4. Discussion

In this study, FUR effectively inhibited cell proliferation in lung cancer cells compared with *β*-ELE, the main component of elemene. *Curcumae Rhizoma *contains much less *β*-ELE than FUR [11]; thus, FUR has potential application as an alternative for *β*-ELE. This study also further clarified the pharmacodynamic material basis for *Curcumae Rhizoma*.

Pharmacological alterations in glycosylation machinery, redox potential, or calcium levels disrupt normal ER protein biogenesis, resulting in ER stress [20]. As a protective mechanism, ER triggers unfolded protein responses to protect cells against ER stress. Therefore, cell death pathways are initiated when ER stress cannot be alleviated [20]. In the present study, FUR upregulated BIP and CHOP expression both in the mRNA and protein levels and accumulated CHOP in cell nuclei, all of which are markers of ER stress [18, 19], after 12 h of treatment. FUR-induced ER stress may trigger a series of cellular responses that lead to proliferative inhibition. However, the detailed mechanism for such an activity requires further study. In addition, we note that the ER stress trigged by FUR is not consistent in different cell lines, which may be due to the different cellular background.

CI is a well-accepted numerical value that provides a qualitative measure of the extent of drug interaction. In this study, CI was used to evaluate the combination effects of FUR and DOX or FUR and TAX on the proliferation of lung cancer A549, NCI-H1299, and 95-D cells. Our data showed that FUR remarkably potentiated TAX-imposed cytotoxicity, as revealed by CI values (Table 1), in NIH-H1299 and 95-D cells. TAX is widely used in clinical settings to treat lung cancers [21, 22]. Further research is recommended to investigate the possible mechanisms involved in such inhibition. It should be aware that the synergetic effects are dependent on the cell types as the synergetic effect is weaker in A549 cells.

In summary, FUR showed anti-proliferative effects similar to or even stronger than those of *β*-ELE and exhibited synergetic effects with TAX in some lung cancer cells. FUR-induced ER stress may partially contribute to its anti-cancer potential, though several detailed mechanisms require further study. These findings indicate that FUR may have the potential to be an anti-cancer natural compound, though there is still a long way to go.

## Figures and Tables

**Table 1:** CI values of FUR at concentrations applied in combination with DOX or TAX in different lung cancer cell lines.

DOX (*μ*M)	TAX (*μ*M)	FUR (*μ*M)	A549		NCI-H1299		95-D	
			CI_(DOX + FUR)_	CI_(TAX + FUR)_	CI_(DOX + FUR)_	CI_(TAX + FUR)_	CI_(DOX + FUR)_	CI_(TAX + FUR)_
0.25	0.025	10	1.96 ± 1.32	0.15 ± 0.07	0.72 ± 0.12	0.17 ± 0.14	1.11 ± 0.61	0.11 ± 0.11
0.5	0.05	20	1.35 ± 0.52	0.40 ± 0.25	1.48 ± 0.15	0.13 ± 0.17	1.63 ± 0.49	0.17 ± 0.21
1	0.1	40	2.38 ± 2.11	0.61 ± 0.16	0.91 ± 0.43	0.16 ± 0.15	1.44 ± 1.04	0.25 ± 0.19
2	0.2	80	0.63 ± 0.12	1.38 ± 1.44	0.40 ± 0.02	0.20 ± 0.12	0.42 ± 0.15	0.35 ± 0.27
4	0.4	160	0.50 ± 0.17	0.66 ± 0.04	0.54 ± 0.07	0.18 ± 0.13	0.74 ± 0.28	0.35 ± 0.29

## Abbreviations

BIP: Binding immunoglobulin protein.
CHOP: C/EBPhomologous protein.
CI: Combination index.
DMSO: Dimethyl sulfoxide.
DOX: Doxorubicin.
*β*-ELE: *β*-elemene.
ER stress: Endoplasmic reticulum stress.
FUR: Furanodiene.
MTT: 3-[4,5-Dimethyl-2-thiazolyl]-2,5-diphenyl tetrazolium bromide.
TAX: Paclitaxel.
